# Targeted Delivery of Mitochondrial Calcium Channel Regulators: The Future of Glaucoma Treatment?

**DOI:** 10.3389/fnins.2017.00648

**Published:** 2017-11-22

**Authors:** Leanne T. Y. Cheung, Abby L. Manthey, Jimmy S. M. Lai, Kin Chiu

**Affiliations:** Department of Ophthalmology, University of Hong Kong, Hong Kong, China

**Keywords:** calcium, mitochondria, glaucoma, mitochondrial drug delivery system, calcium channel blockers

## Introduction

Glaucoma is a multifactorial neurodegenerative disease affecting 64.3 million people worldwide (Tham et al., 2014). Despite vigorous research on new treatments, those that reduce intraocular pressure (IOP) remain the gold standard. However, their effectiveness has been questioned as they only slow down degeneration without significantly reversing or stopping the disease (Osborne et al., 2016b). Recent studies have, therefore, investigated the causative roles of other processes, including glutamate toxicity, glial overactivation, etc., (Mann et al., 2005; Chong and Martin, 2015; Lopez Sanchez et al., 2016; Vecino et al., 2016).

Mitochondrial dysfunction is another widely studied causal process in the development of glaucoma and has also been investigated as a potential drug target. For example, red light therapy, manipulation of the mammalian target of rapamycin (mTOR) pathway, and nicotinamide treatment are three recently investigated clinical therapies for glaucoma-related mitochondrial dysfunction (Osborne et al., 2016a,b; Williams et al., 2017). Mitochondrial activity is intimately linked to oxidative metabolism and reactive oxygen species (ROS) formation (Schieke et al., 2006). ROS production is known to cause retinal ganglion cell (RGC) apoptosis and subsequent vision loss. Furthermore, while mitochondrial function is regulated by multiple pathways, calcium signaling likely plays a key role (Vosler et al., 2008; Hurst et al., 2017). In fact, plasma membrane calcium channel inhibitors were recently found to arrest acute axonal degeneration and improve regeneration after optic nerve crush (Ribas et al., 2017). A different combination of calcium permeability inhibitors also preserved optokinetic reflex following partial optic nerve transection (Savigni et al., 2013). While the inhibitors utilized in these studies targeted calcium channels in the plasma membrane, their effects indicate that ROS generation and calcium signaling, which are significantly regulated by the mitochondria, are critical during glaucoma pathogenesis.

**Figure 1:**
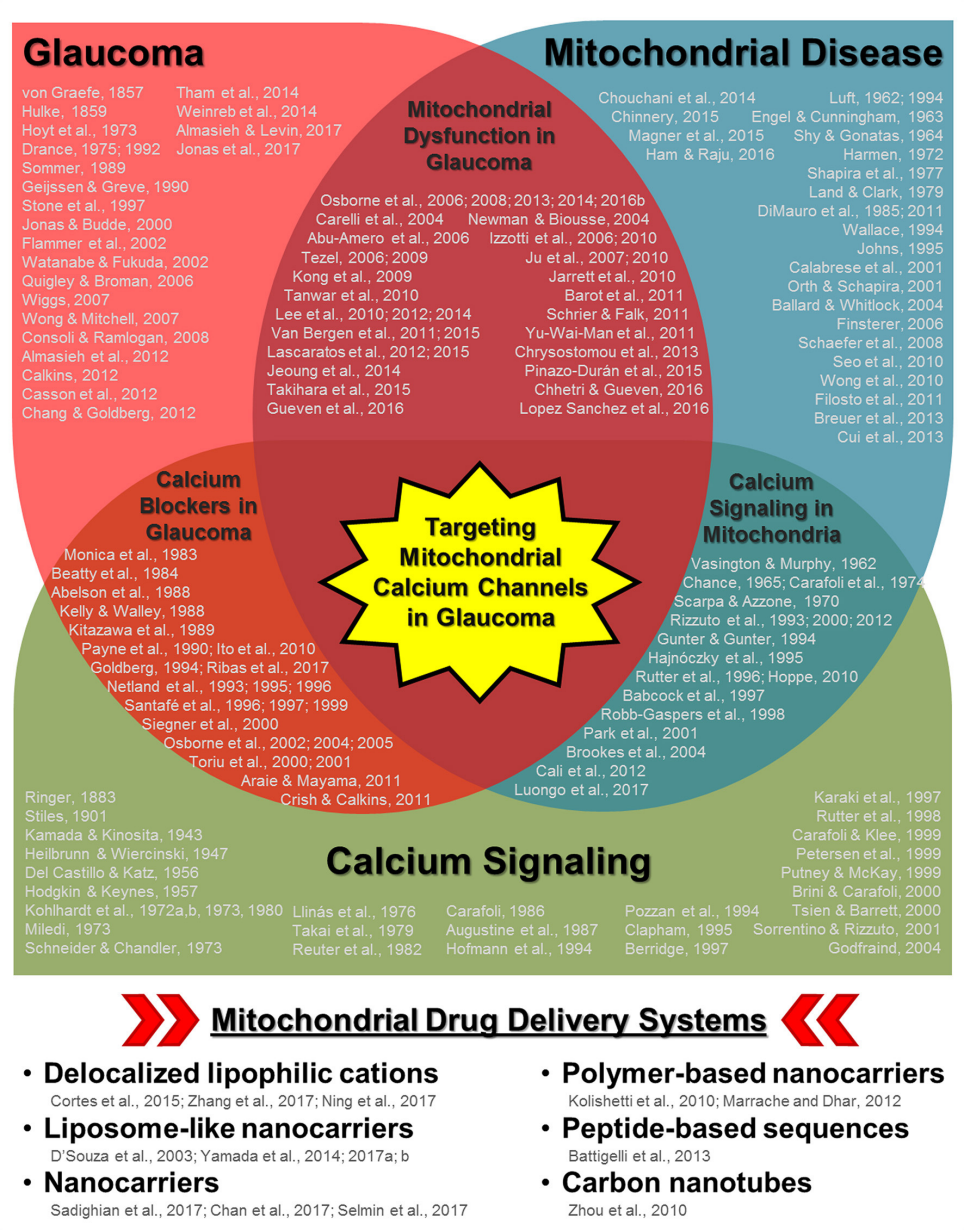
Schematic diagram highlighting the relationships between glaucoma, mitochondrial disease/aging, and calcium signaling along with multiple keystone studies and reviews from prominent research groups. Some of the earliest published research concerning disease pathology and mechanisms are listed for each respective field as well as in areas of overlap (e.g., mitochondrial dysfunction in glaucoma, calcium channel treatment in glaucoma, and calcium signaling in mitochondria). While it is not possible to list all of the influential research published in each field, those listed include some of the key historical publications, with particular emphasis on relationships with the ocular environment or neurodegeneration when applicable. The cumulative research reported in these publications (and those cited within) in each respective field as well as other disease contexts has led to the development of multiple mitochondria-specific drug delivery systems, listed in the bottom panel. Their validation in parallel with the continued investigation of mitochondrial calcium signaling during disease pathogenesis indicate that targeting mitochondrial calcium channels during glaucoma could be a powerful therapeutic tool.

Recently, a mitochondrial-specific drug delivery system was shown to be effective in increasing drug concentration in mitochondria in hepatic injuries and drug-resistant cancer cells (Yamada and Harashima, 2017; Yamada et al., 2017). However, the full potential of this system (and other similar systems) has not been fully evaluated with regards to calcium regulation in the diseased retina. In this opinion article, we provide a brief discussion concerning the role of mitochondrial calcium regulation during glaucoma pathogenesis as well as insight concerning the potential use of mitochondrial-specific drug delivery during disease treatment. We believe that the extensive research and overlap in the fields of glaucoma and mitochondrial disease/aging (including calcium signaling dysfunction) ultimately lead to the therapeutic utilization of mitochondrial-specific delivery of calcium channel regulators during glaucoma and other retinal/neurodegenerative diseases (Figure 1).

## Glaucoma pathophysiology

Glaucoma is a two phase degenerative disease. The first phase involves a primary insult to the RGCs (Levkovitch-Verbin et al., 2003). Confirmed risk factors/insults for glaucoma include high IOP, ischemia, and aging. While these direct insults have classically been investigated as the cause of glaucoma-related vision loss, recent evidence indicates that damage to the visual cortex and/or optic nerve (i.e., distal axonopathy), which is then propagated to the retina following stress on axonal transport systems, may play a significant role in the initiation of the disease (Calkins and Horner, 2012; Crish and Calkins, 2015). Ultimately, all of these insults disrupt oxygen supply and alter retinal function. Furthermore, mitochondrial oxidative phosphorylation is significantly less effective in the affected RGCs, and energy production depends more on glycolysis and the tricarboxylic acid cycle. This change in energy supply causes oxidative stress and reduced ROS consumption, leading to mitochondrial damage and further ROS accumulation (Nguyen et al., 2011). While it has been hypothesized that RGCs can still function normally in this reduced energy state (Osborne et al., 2016b), they are more susceptible to secondary insults.

Secondary affronts to the RGCs can come in various forms. For example, primary insult-induced activation of retinal microglia and astrocytes as well as altered Müller cell function have detrimental secondary consequences related to the release of pro-inflammatory markers as well as other cytotoxic substances, including glutamate, nitrogen oxide, etc., in the extracellular space surrounding the RGCs. Furthermore, aged/dysfunctional mitochondria within the RGCs can also act as secondary stressors. In aged mitochondria, the initial increase in oxidative stress and reduced ROS consumption is amplified, resulting in a vicious positive feedback loop involving ROS along with damage to mitochondrial and nuclear DNA (Nguyen et al., 2011). This damage is largely irreversible as the repair mechanisms are often impaired in aged mitochondria. ATP production in cells with damaged mitochondria also becomes increasingly more difficult, ultimately leading to calcium dysregulation. As calcium is a known trigger for glutamate release (Neher and Sakaba, 2008), disrupted calcium signaling in aged mitochondria can further exacerbate primary insult-induced glutamate toxicity. Interestingly, increased glutamate concentration also mediates calcium influx (Wojda et al., 2008), indicating multiple points of crosstalk between mitochondrial calcium signaling and neuronal function.

## Mitochondrial calcium as a key player in glaucoma

Mitochondria have two membrane layers. At the outer membrane, calcium influx is largely mediated through voltage-dependent anion channels (VDACs) (Cali et al., 2012; Rizzuto et al., 2012). Some reports suggest that open-state VDACs facilitate metabolite flow and prevent cytochrome C release, while closed-state VDACs mediate the opposite (Tan and Colombini, 2007; Hoppe, 2010; Williams et al., 2013). Further, elevated intracellular calcium concentrations appear to increase VDAC1 oligomerization and downstream apoptosis (Keinan et al., 2013). This increase in oligomerization has been demonstrated to be mediated specifically by mitochondrial, rather than cytosolic, calcium, providing a direct link between mitochondrial calcium and apoptosis.

At the inner membrane, calcium influx from the intermembrane space into the matrix is largely regulated by calcium-activated mitochondrial calcium uniporters (MCUs) (Cali et al., 2012). Although MCU calcium affinity is low, their effect on calcium concentration is significant as they mediate calcium inflow in response to the negative membrane potential/calcium gradient created by pumping protons across the membrane during oxidative phosphorylation. Thus, MCUs are functionally dependent on both intracellular calcium concentration and energy demand (Tsai et al., 2017). The links between calcium and energy are further strengthened by the calcium-dependent activation of three metabolic enzymes, pyruvate, α-ketoglutarate, and isocitrate dehydrogenases, all of which function in the tricarboxylic acid cycle (Cali et al., 2012). MCU function also depends on the proximity of the mitochondria to other calcium regulating organelles, including the endoplasmic reticulum, sarcoplasmic reticulum, and plasma membrane, which can alter the local calcium concentration (Kirichok et al., 2004; Rizzuto et al., 2012).

Cellular calcium homeostasis, whereby nanomolar levels of free calcium are found in the cytosol, is maintained, at least in part, via effective buffering mechanisms, including pH and phosphate/adenosine availability. Additional intermitochondrial buffering mechanisms involve calcium efflux via electrogenic Ca^2+^/3Na^+^ exchangers (mNCXs) and/or electroneutral Ca^2+^/2H^+^ exchangers (mHCXs) located on the inner mitochondrial membrane. mNCXs are the main efflux channels in excitable tissues, including RGCs, while mHCXs are found mainly in non-excitable tissues (Hoppe, 2010). Interestingly, these efflux systems appear to change into influx pathways during glaucoma (Wojda et al., 2008). In aged neurons, the expression of calcium buffering proteins, including calbindin-D28k, calretinin, and parvalbumin, is also reduced (Bu et al., 2003). Together, these changes in efflux levels and calcium buffering protein expression significantly alter calcium gradients, cytosolic calcium levels (Williams et al., 2013), and mitochondrial membrane polarization (Wojda et al., 2008), resulting in altered/inefficient oxidative phosphorylation, ROS accumulation, downstream changes in mitochondrial function, and neuronal cell survival.

In retinal neurons, intracellular free calcium overload triggers calpain activation which can subsequently initiate apoptotic cascades (Sharma and Rohrer, 2004; Huang et al., 2010; Kar et al., 2010). Calpain is a calcium-dependent cysteine protease that, once activated, cleaves pro-apoptotic B-cell lymphoma (Bcl)-2 family members as well as apoptosis-inducing factor (AIF) and inner membrane mNCXs (Vosler et al., 2008). Calpain activation is also related to the formation and opening of mitochondrial permeability transition pores (mPTPs) (Cali et al., 2012; Bernardi and Di Lisa, 2015). mPTPs are multi-protein complexes that facilitate calcium efflux. However, unlike mNCXs, once the mPTPs are opened, the inner membrane is irreversibly permeabilized, resulting in uncontrolled dissipation of the electrochemical gradient, ATP depletion, ROS production, cytochrome C efflux, and mitochondrial swelling (Rasheed et al., 2017). Notably, increased cytoplasmic cytochrome C levels not only facilitate additional calpain activation, but the coupling of cytochrome C with apoptosis protease-activating factor (APAF)-1 results in apoptosome formation. Apoptosomes recruit and activate caspase-9 and downstream caspase-mediated apoptosis. Interestingly, activation of various caspases also feeds back into the process to further activate pro-apoptotic Bcl-2 proteins family members and increase mitochondrial permeability.

## Calcium-related drug treatments in the ocular environment

Various calcium regulating therapies have been investigated for their use in treating visual neurodegeneration (Kamel et al., 2017). Indeed, in the dorsal lateral geniculate nucleus and superior colliculus as well as RGCs, lomerizine, a well-known plasma membrane calcium channel blocker, has been used to manage neuronal degeneration (Ito et al., 2010; Selt et al., 2010). Various combinations of calcium channel inhibitors, including the L-/N-type channel blocker amlodipine, T-type channel blocker amiloride, α-amino-3-hydroxy-5-methyl-4-isoxazolepropionic acid (AMPA) receptor blockers, and purinergic receptor blockers, also increase RGC survival, reduce axonal degeneration, and increase axonal regeneration in both partial optic nerve transection (Savigni et al., 2013) and crush models (Ribas et al., 2017). However, the effects on visual function preservation were not significant.

In glaucoma and retinitis pigmentosa models, inhibition of calpain signaling has also been demonstrated to be beneficial for RGC and photoreceptor survival, respectively. This is not surprising as the detrimental effects of calcium overload are mediated largely through calpain activation. Latanoprost, an ocular anti-hypertension drug, for example, modulates its neuroprotective effects in this calpain-mediated manner (Yamamoto et al., 2017).

Unfortunately, beyond these studies using agents against calcium signaling in the plasma membrane, other mitochondria-specific calcium channel regulators to block detrimental calcium changes have not been intensively studied and none have been investigated as a glaucoma treatment option. Notably, calcium can be transported across the outer and inner mitochondrial membranes via VDACs and MCUs, respectively, as well as during unregulated diffusion through mPTPs, making each of these protein complexes a potential target for calcium regulation during disease. While a number of agents have been used to block mPTPs (Kajitani et al., 2007; Halestrap and Richardson, 2015), treatment with these agents leaves the causative upstream changes in calcium concentration and channel function largely unchecked. Thus, targeting VDACs and/or MCUs and avoiding mPTP formation altogether would potentially be more advantageous. For example, an anti-VDAC antibody has been shown to reduce cytochrome C release from mitochondria (Madesh and Hajnóczky, 2001). Unfortunately, directly altering VDAC function in this manner can also manipulate the transport of other essential metabolites (Camara et al., 2017). In cancer, blocking VDACs results in apoptosis (Shoshan-Barmatz et al., 2017), which is counterproductive to the cellular rescue required during glaucoma treatment. Alternatively, an MCU blocker, Ru360, was demonstrated to alter ion transport through these channels as well as block iron overload and has the advantage of having minimal effects on other cellular functions (Sripetchwandee et al., 2013). This same blocker was also previously shown to prevent the accumulation of mitochondrial free calcium despite high cytosolic free calcium concentrations in post-ischaemic rat heart cells (de Jesús García-Rivas et al., 2005). Lastly, this drug also maintains normal oxidative phosphorylation levels and prevents mPTP opening, while other organelles and cellular processes are unaffected, making it a drug of interest for glaucoma therapy.

## Mitochondrial-specific drug delivery as a means to treat glaucoma

Organelle-specific drug targeting itself is not novel, being reviewed in multiple excellent publications (Sakhrani and Padh, 2013; Zhang and Zhang, 2016). While mitochondrial-specific drug targeting has not been applied to glaucoma, researchers have been actively proposing new mitochondrial delivery/transporter systems to target this organelle in other diseases. For example, a liposomal-based carrier was recently described that uses octaarginine modification, electrostatic attraction, and membrane fusion to promote mitochondrial uptake (Yamada and Harashima, 2017). This style of “MITO-porter” was then used to deliver coenzyme Q10 in mice with hepatic ischemic/reperfusion injuries and mediated a significant decrease in serum alanine aminotransferase (ALT) (Yamada et al., 2015). Another study utilized a MITO-porter system to target doxorubicin to the mitochondria of drug-resistant cancer cells, successfully destroying these cells (Yamada et al., 2017). Other nanotechnology techniques have also been employed, including a recent hybrid of polylactide-co-glycolide nanoparticles and mitochondria-penetrating particles (Selmin et al., 2017; Figure 1, bottom panel). Taken together, the evidence emerging from these investigations provides a solid foundation for the continued study of these delivery systems in other cellular contexts.

Delivery of agents used to modulate channel function along with the expression of other essential compounds (e.g., cytochrome C, ATP, etc.,) in concentrated amounts directly to the mitochondria during glaucoma using these systems would allow some of the downstream detrimental changes to be managed before vision loss. While some current (e.g., Ru360) and future drugs targeting mitochondrial calcium channels already innately target the mitochondria, the use of MITO-porters and similar delivery systems would not only allow higher concentrations to be delivered, but would also avoid any unknown effects on other organelles. Furthermore, delivery systems could also be used to package multiple drugs/compounds together in order to have the greatest therapeutic effect. Ultimately, these drugs would collectively reduce calcium efflux and restore calcium homeostasis as well as prevent mPTP formation, ATP depletion, ROS production, and cytochrome C dissipation. Doing so would prevent the second wave of apoptosis, allowing the cells to function normally even after the initial insult. While these drug delivery systems are not currently used as an ocular disease treatment, their potential to transport drugs to the retina and/or optic nerve/visual cortex that will subsequently manipulate mitochondrial function is a promising research avenue for novel treatment development for glaucoma as well as other retinal pathologies.

## Conclusions

Mitochondrial dysfunction and the associated changes in calcium homeostasis, ROS production, and energy supply are intimately related to RGC death/dysfunction during glaucoma, making it an attractive treatment target. Mitochondrial-targeting drug delivery systems, which have been developed and validated in other cellular environments, could potentially avoid these issues by packaging multiple drugs and delivering them at high concentrations directly to the mitochondria. In discussing the recent advances in these techniques within the context of mitochondrial calcium regulation during glaucoma for the first time, we pose the question: Is this the future of glaucoma treatment? The relationships highlighted in multiple keystone studies investigating glaucoma and mitochondrial disease/aging in addition to the essential role of calcium signaling in these processes indicate an affirmative answer. Thus, while the full potential of these systems has yet to be fully established, we believe that mitochondrial-specific delivery of calcium channel regulators could effectively change how glaucoma and other neurodegenerative diseases affecting the retina are treated.

## Author contributions

All authors listed have made a substantial, direct and intellectual contribution to the work, and approved it for publication.

### Conflict of interest statement

The authors declare that the research was conducted in the absence of any commercial or financial relationships that could be construed as a potential conflict of interest.
